# Postnatal CMV Infection in Extremely Premature Newborns: A Single-Center Exploratory Study

**DOI:** 10.3390/children13050703

**Published:** 2026-05-20

**Authors:** Rok Košiček, Vanja Erčulj, Lilijana Kornhauser Cerar, Lev Bregant, Miroslav Petrovec, Marko Pokorn, Ana Spirovska, Tina Uršič, Urška Rahne Potokar, Stefan Grosek

**Affiliations:** 1 Department of Anaesthesiology, Celje General Hospital, 3000 Celje, Slovenia; rok.kosicek@sb-celje.si; 2 Faculty of Criminal Justice and Security, University of Maribor, 2000 Maribor, Slovenia; vanja.erculj@um.si; 3 Rho Sigma, 1000 Ljubljana, Slovenia; 4 Neonatal Intensive Care Unit, Department of Perinatology, Division of Gynaecology and Obstetrics, Uuniversity Medical Centre Ljubljana, 1000 Ljubljana, Slovenia; lilijana.kornhauser@kclj.si (L.K.C.); lev.bregant@guest.arnes.si (L.B.); ana.spirovska@kclj.si (A.S.); 5 Institute of Microbiology and Immunology, Faculty of Medicine, University of Ljubljana, 1000 Ljubljana, Slovenia; mirc.petrovec@mf.uni-lj.si (M.P.); tina.ursic@mf.uni-lj.si (T.U.); 6 Division of Paediatrics, University Medical Centre Ljubljana, 1000 Ljubljana, Slovenia; marko.pokorn@kclj.si; 7 Department of Paediatrics, Faculty of Medicine, University of Ljubljana, 1000 Ljubljana, Slovenia; 8 Slovenian Institute for Transfusion Medicine, 1000 Ljubljana, Slovenia; urska.rahne@ztm.si

**Keywords:** postnatal CMV, extremely premature newborns, breast milk, thrombocytopenia, leukopenia

## Abstract

**Highlights:**

**What are the main findings?**

- Postnatal CMV infection in extremely premature newborns was associated with increased incidence of hepatosplenomegaly.
- Postnatal CMV infection in extremely premature newborns was associated with lower platelet and leukocyte counts.

**What are the implications of the main findings?**

- Postnatal CMV infection is associated with a more severe clinical course of extremely premature newborns.
- More attention should be paid to preventing CMV transmission to extremely premature newborns.

**Abstract:**

Background/Objectives: The impact of postnatal CMV infection in extremely premature newborns is poorly characterized. We aimed to determine the impact and outcomes of postnatal CMV infection in a population of extremely premature newborns of gestational age of less than 29 weeks hospitalized in the Department of Perinatology, Division of Gynecology, University Medical Center Ljubljana. Methods: We included all extremely premature newborns of gestational age of less than 29 weeks treated in the Department of Perinatology between December 2022 and December 2024. Newborns were screened for CMV infection at set timepoints and congenital infection was excluded with PCR testing. Additional PCR testing for CMV was performed if newborns developed clinical features suspect for postnatal CMV infection. Clinical characteristics and treatment outcomes of newborns were noted. Mothers of infected newborns had their CMV serostatus determined. Results: In total, 63 extremely premature newborns were included, and 14 newborns had confirmed postnatal CMV infection. Postnatal CMV infection was associated with hepatosplenomegaly and lower platelet and leukocyte counts compared to controls. We found no association between postnatal CMV infection and other neonatal comorbidities. Conclusions: In our study, postnatal CMV infection was associated with a more severe and prolonged clinical course of extremely premature newborns compared to uninfected controls; however, in multivariable analysis, the association with a prolonged length of stay was no longer statistically significant. The results suggest that preventing postnatal CMV infection in this population is important.

## 1. Introduction

Cytomegalovirus (CMV) infection in newborns can be congenital (cCMV) or postnatal (pCMV). While cCMV is increasingly recognized as an important cause of morbidity in newborns, pCMV is less studied and its impact is less clear [1]. It appears that pCMV has less impact in term newborns or preterm newborns with gestational age ≥ 32 weeks [2], perhaps because of a significantly higher placental transfer of IgG anti-CMV antibodies after 34 weeks of gestation [3].

It is well established that one of the most common modes of pCMV transmission to a newborn is by the mother’s milk [4,5,6,7,8,9]. Global CMV seroprevalence is estimated to be around 86% in women of childbearing age [10]. An important study by Hamprecht et al. showed that CMV reactivation in seropositive breastfeeding mothers is extremely common and viral shedding in breast milk in these mothers is also common, leading to CMV transmission in 34% of preterm newborns [11]. Another study by Martins-Celini et al. found a similarly high incidence of CMV reactivation in seropositive mothers, with a lower incidence of transmission, but their results showed that the rate of transmission was inversely related to gestational age [12]. A meta-analysis including 2502 newborns evaluated the incidence of breast milk-acquired pCMV in preterm newborns born to CMV-seropositive mothers and reported the incidence of CMV shedding into the breast milk of 80.5% of CMV-seropositive mothers, with CMV transfer to breastfed premature newborns of CMV-seropositive mothers occurring in 16.5% [6].

In addition to the clinical presentation of pCMV, which includes sepsis-like symptoms, hepatitis, bone marrow suppression and pneumonitis [13], pCMV in previous studies was also associated with necrotizing enterocolitis (NEC) [14,15], bronchopulmonary dysplasia (BPD) [16], retinopathy of prematurity (ROP) [12], and sensory neural hearing loss (SNHL) [17]. Our study group previously conducted a 12-year retrospective study on newborns of <29 weeks of gestational age who were tested for pCMV. In that study, we could not confirm any associations between pCMV and treatment outcomes, but because of the study design, these could not be ruled out [18]. However, length of stay, hearing outcomes, and neurodevelopmental outcomes were broadly similar across the three groups: pCMV-negative infants, pCMV-positive infants who did not receive antiviral treatment, and pCMV-positive infants who received antiviral treatment. In contrast, most recent studies in very preterm infants with pCMV infection have reported longer hospital stays and increased need for respiratory support, while some have also identified higher risks of bronchopulmonary dysplasia, growth restriction, hearing impairment, and neurodevelopmental delay [17,19,20,21,22,23].

In a previous study, the prevalence of cCMV in Slovenia was low (0.14%) [24], but the epidemiology of pCMV in the at-risk population of extremely premature newborns in Slovenia has not yet been characterized. There is also a protocol in place in Slovenia for the management of cCMV, but not for pCMV [25].

We aimed to characterize the length of stay and outcomes of pCMV in a population of extremely premature newborns of <29 weeks of gestational age in the Department of Perinatology, Division of Gynecology, University Medical Center Ljubljana, and assess associations between CMV infection and the clinical characteristics of these newborns. We also determined the serostatus of mothers of infected newborns to better characterize CMV transmission and clinical course. 

## 2. Materials and Methods

### 2.1. Study Design

We conducted an exploratory, prospective, single-center observational study of extremely premature newborns of gestational age less than 29 weeks.

For this study, we used a clinical pathway for CMV infection in this population. Newborns were grouped according to their pCMV status into pCMV-positive (pCMV+) and pCMV-negative (pCMV−) groups. All newborns of gestational age less than 29 weeks were PCR tested for CMV in a urine sample in the first 3–5 days after birth to rule-out cCMV. All newborns were again tested for CMV at 21 days after birth. We also tested them for CMV infection if, at any time during hospitalization, they developed clinical signs consistent with CMV infection that could not otherwise be explained (hepatomegaly and/or splenomegaly, elevated liver tests, sepsis-like syndrome, new respiratory failure, thrombocytopenia). Postnatal CMV infection (pCMV) was defined as the detection of CMV at ≥21 days after birth in conjunction with negative CMV PCR results in the first 3–5 and before 21 days after birth.

Newborns with a severe clinical course of pCMV (sepsis-like syndrome, CMV pneumonitis) were treated with antivirals after consultation with a pediatric infectious disease specialist. Untreated newborns with pCMV were closely monitored clinically and with laboratory tests.

To determine the association between pCMV infection in newborns and CMV serostatus and seroconversion in their mothers, we determined the serostatus of mothers of pCMV-positive newborns, during pregnancy and at the time of pCMV diagnosis. Primary CMV infection in a mother during pregnancy was defined as the presence of IgM and low-avidity IgG anti-CMV antibodies. Reactivation of latent CMV infection in a mother (i.e., seroconversion) was defined as the presence of IgM and high-avidity IgG anti-CMV antibodies [26].

### 2.2. Participants

Extremely premature newborns of gestational age less than 29 weeks who were hospitalized in the neonatal intensive care unit (NICU) at the Division of Gynecology and Obstetrics, University Medical Center (UMC) Ljubljana, Slovenia, between December 2022 and December 2024 were included.

Exclusion criterion was a length of hospital stay of less than 21 days.

### 2.3. Microbiological Methods

Part of the CMV UL123 gene was detected in urine samples using GeneProof quantitative real-time PCR, performed according to the manufacturer’s instructions (GeneProof, Brno, Czech Republic). Nucleic acids were automatically extracted from 200 µL of urine samples and eluted in 90 µL, using an EZ1&2 Virus Mini Kit v2.0 on an EZ2 Connect instrument (QIAGEN, Hilden, Germany). GeneProof quantitative real-time PCR has a validated linear dynamic range between 10 and 10,000 IU/µL of a sample. In our laboratory, the limit of detection (LoD) was established at approximately 0.5–1 IU/µL. Samples with viral loads within the linear range were considered reliably quantifiable, while samples with a detectable viral load below this range were interpreted as positive but not accurately quantifiable. CMV serostatus and seroconversion from serum or plasma samples in mothers were determined using the LIAISON^®^ CMV IgG II CLIA assay for quantitative determination of specific IgG antibodies and the LIAISON^®^ CMV IgM II CLIA assay for semi-quantitative determination of specific IgM antibodies, on a LIAISON^®^ XL Analyzer, according to the manufacturer’s instructions (DiaSorin S.p.A., Saluggia, Italy) or the ARCHITECT CMV IgG CMIA assay for quantitative determination of specific IgG and ARCHITECT CMV IgM CMIA assay for qualitative determination of specific IgM antibodies on an ARCHITECT analyzer (Abbott Laboratories, Abbott Park, IL, USA). All assays were performed according to the manufacturer’s instructions.

### 2.4. Clinical Characteristics

The clinical characteristics of newborns and their mothers were obtained from discharge notes, electronic medical records and medical charts. We collected laboratory markers, clinical signs and vasopressor and ventilatory needs at pCMV diagnosis (in the pCMV+ group) or at the age of 21 days after birth (in the pCMV− group), unless otherwise stated. We allowed a deviation of 7 days from this time frame for laboratory markers only.

The assessed maternal clinical characteristics were: CMV serostatus during pregnancy and at the time of CMV infection detection in their newborn, and the presence of preeclampsia, eclampsia or diabetes.

The assessed newborn clinical characteristics were: demographic data (gestational age, birth weight, birth length, head circumference at birth, small for gestational age (SGA) status); Apgar scores at 1 and 5 min; breastfeeding status; presence of hepatomegaly and/or splenomegaly, petechiae, jaundice and sepsis-like syndrome; laboratory markers (aspartate aminotransferase (AST), alanine aminotransferase (ALT), gamma-glutamyl transferase (γ-GT), C-reactive protein (CRP), and platelet, leukocyte and neutrophil counts), antiviral therapy for CMV infection (duration and drugs received); dexamethasone course during hospitalization or diuretics during hospitalization; vasopressor needs, FiO2 and type of ventilatory support at CMV infection diagnosis; comorbidities (periventricular leukomalacia (PVL), bronchopulmonary dysplasia (BPD), retinopathy of prematurity (ROP), necrotizing enterocolitis (NEC) and stage 3 or 4 intraventricular hemorrhage (IVH)); transitory evoked otoacoustic emissions (TEOAEs) at discharge; length of stay; and death during hospitalization. The newborns in our study were fed both fresh and frozen–thawed mother’s milk.

SGA was defined as birth weight lower than the 10th percentile for gestational age based on Slovenian growth charts. Hepatosplenomegaly was defined as the liver extending 2 cm below the right costal margin and the spleen extending below the left costal margin. Jaundice was defined as yellow pigmentation of the skin by clinical examination and increased bilirubin levels. The presence of hepatosplenomegaly and jaundice was discerned from daily physical examination notes. Sepsis-like syndrome was defined as a combination of gray skin pallor, episodes of apnea and bradycardia. IVH was staged according to Papile [27].

Length of stay (LOS) in the neonatal intensive care unit (NICU) was defined as the period from admission to the NICU after birth until transfer to the mother’s ward (rooming-in), once the infant was able to bottle-feed for 48–72 h and cardiorespiratory parameters were stable.

### 2.5. Outcomes

The primary outcome was length of stay in the intensive care unit until stepping down to the ordinary ward. Secondary outcomes were incidences of important neonatal morbidities (PVL, BPD, ROP, NEC, IVH), non-responsiveness of TEOAEs at discharge and death before discharge.

### 2.6. Ethical Considerations

This study was approved by the National Medical Ethics Committee of the Republic of Slovenia (ref. no. 0120-433/2022/6). Informed consent was obtained from mothers of CMV-positive newborns, whose blood was analyzed for CMV antibodies.

### 2.7. Statistical Analysis

Categorical variables were described with frequencies and percentages, and numerical variables, due to a small cohort of CMV-positive newborns, with medians and interquartile ranges (IQRs). The association between several factors and postnatal CMV status was tested using univariate logistic regression or the likelihood ratio test in the event of zero cases in one of the categories. The Apgar score was treated as a numerical predictor, which is a common approach when the scale has several ordered categories and is approximately linear in relation to the log-odds of the outcome. Because of the small number of CMV-positive newborns, the applicability of multivariable analyses was limited and the risk of model overfitting was considered. Therefore, the primary analyses were based on univariate models. Additional adjusted logistic regression analyses were performed exploratorily to assess the robustness of selected associations in relation to clinically relevant or group-differing covariates, while restricting the number of adjustment variables. Statistical analysis was performed by SPSS version 29. The significance level was set at α = 0.05. No correction for multiple testing was applied.

## 3. Results

**Figure 1:**
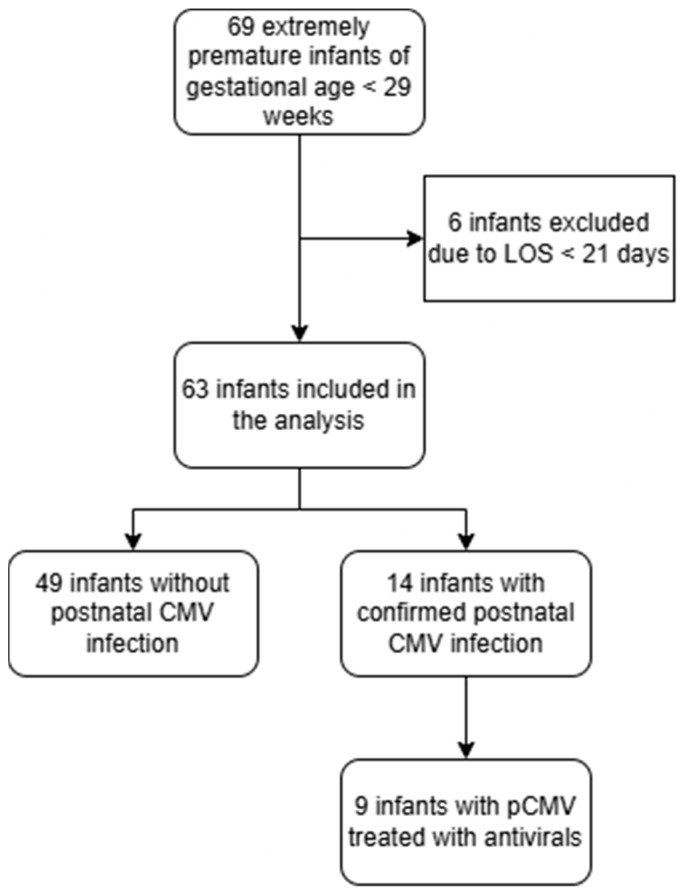
Flow chart of our study participants.

Sixty-nine extremely premature newborns with a gestational age < 29 weeks were born in the Department of Perinatology, Division of Gynecology, University Medical Center Ljubljana (UMC Ljubljana), between December 2022 and December 2024 and hospitalized in the NICU UMC Ljubljana, which represented approximately more than 76% of all premature newborns with less than 29 weeks gestational age born in Slovenia. Six newborns were excluded from the study due to a LOS of less than 21 days. Of the 63 newborns included in the analysis, 14 had confirmed pCMV and 49 were uninfected (Figure 1). All newborns had cCMV ruled out with negative CMV PCR from a urine sample in first 3–5 days and at 21 days of life.

The newborns’ characteristics at birth—overall and by postnatal CMV status—are summarized in Table 1. There were 35 (56%) males. The median (IQR) gestational age of newborns was 25 (24–27) weeks and median (IQR) birth weight was 750 (650–950) g. The median (IQR) length at birth was 33 (32–35) cm. The median (IQR) head circumference was 23.5 (22–24.5) cm. At least half of the newborns had an Apgar score at 1 min of six or more (IQR: 4–7) and at 5 min of seven or more (6–8). The majority (59; 94%) of newborns were fed mother’s milk. Analysis of the association between newborns’ characteristics at birth and CMV status showed a statistically significant association between length at birth and CMV status. Children with postnatal CMV were smaller (median (IQR): 32 (30–33 cm)) in comparison to newborns that remained CMV-negative (median (IQR): 33 (32–36 cm)). No association was found between other characteristics at birth and CMV status.

Clinical characteristics and treatment outcomes of newborns included in the study—overall and by postnatal CMV status—are summarized in Table 2. A statistically significant association between splenomegaly (*p* < 0.001), hepatomegaly (*p* < 0.001), hepatosplenomegaly (*p* < 0.001), and postnatal CMV status was found. A statistically significantly higher share of newborns with postnatal CMV had splenomegaly (5; 36%), hepatomegaly (4; 29%), and hepatosplenomegaly (4; 29%). In CMV-negative newborns, only one child had splenomegaly, while there were no newborns with hepatomegaly, hepatosplenomegaly or jaundice. LOS was significantly lower (*p* = 0.014) in CMV-negative newborns (Me (IQR): 71 (58–94)) than in CMV-positive newborns (Me (IQR): 111 (66–142)). Cause of death was *S. aureus* sepsis in combination with NEC in one newborn and toxic shock in combination with NEC in another newborn.

Gestational age was considered the most relevant clinical potential confounder for neonatal length of stay. However, since gestational age was comparable between groups, while length at birth differed significantly and was strongly correlated with gestational age, an additional exploratory adjustment analysis using length at birth as a covariate was performed. In this analysis, associations between pCMV and hepatosplenomegaly (OR = 19.1; 95% CI: 1.9–189.8; *p* = 0.012), leukocyte count (OR = 0.74; 95% CI: 0.59–0.92; *p* = 0.018) and platelet count (platelets: OR = 0.99; 95% CI: 0.99–1.00; *p* = 0.015) remained statistically significant, but the association between pCMV and LOS was not anymore statistically significant after the adjustment (OR = 1.01; 95% CI: 0.69–1.19; *p* = 0.136).

The newborns’ laboratory results—overall and by postnatal CMV status—are summarized in Table 3. A statistically significant association between platelet and leukocyte counts and postnatal CMV status was found. Newborns with higher values of both laboratory parameters had lower odds for postnatal CMV.

Seven (12%) mothers had preeclampsia and five (8%) had diabetes in pregnancy. No association between either of the two pregnancy complications and newborns’ postnatal CMV was found. Blood samples were taken from 10/11 mothers whose newborns had postnatal CMV. Half of the samples were taken until 58 days after birth (IQR: 46–67). Seroconversion was found in three blood samples.

None of the newborns had CMV detected in samples collected at 3–5 days or 21 days after birth. Postnatal CMV was found in 14 (22%) newborns. Of those, nine newborns received antiviral treatment. The median (IQR) treatment duration was 35 (23–38) days. Median (IQR) age at diagnosis was 59 (46–67) days. All of the treated newborns were initially treated with ganciclovir and seven out of nine newborns continued treatment with valganciclovir. Three newborns were additionally treated with intravenous immunoglobulins containing antibodies against the cytomegalovirus. Median (IQR) LOS was 127 (115–146) days in pCMV-positive newborns who were treated with antivirals compared to 59 (56–66) days in untreated newborns (data are not presented).

Newborns in the postnatal CMV cohort that did not receive treatment were older, longer and had a higher birth weight in comparison to newborns that received treatment.

IgG anti-CMV antibodies were detected in nine mothers of 12 pCMV-positive newborns, one mother of a pCMV-positive newborn was not tested, and one mother of a pCMV-positive newborn was CMV-seronegative. Seroconversion was confirmed in the mothers of two out of three newborns that did not receive treatment and in the mothers of one out of seven newborns that received treatment.

## 4. Discussion

Newborns in the pCMV-positive group had a significantly lower length at birth compared to pCMV-negative controls, but there were no differences between both groups in other newborn characteristics at birth. This could be interpreted as lower length at birth being a risk factor for pCMV infection.

Among pCMV-positive newborns, we found a statistically significant higher incidence of hepatosplenomegaly and jaundice and statistically significant lower platelet and leukocyte counts compared to pCMV-negative controls. The length of stay in the neonatal intensive care unit was also significantly longer in the univariate logistic regression analysis; however, this association was no longer statistically significant after adjustment in the multivariate logistic regression model using birth length as a control variable. In contrast, the associations between pCMV and hepatosplenomegaly, leukocyte count and platelet count remained statistically significant after multivariate adjustment. No association was found between pCMV infection and the incidence of major neonatal comorbidities (BPD, ROP, NEC, IVH, PVL).

Previous studies have shown conflicting results concerning clinical outcomes in extremely premature newborns with pCMV. Some have found worse short-term and long-term outcomes of infected newborns [12,16,17,23,28,29,30,31], while others have not [18,32,33,34,35]. A recent study by Lee et al. concluded that pCMV in premature newborns mostly presents with a favorable clinical course and outcomes, but newborns with a more severe clinical course who were treated with antivirals had a significantly lower mean gestational age than those untreated [35]. In our study, the median gestational age of all included newborns was 25 weeks, which could explain why pCMV was associated with a more severe clinical course. Similar to the findings of Lee et al., the newborns that were treated with antivirals in our study also had an even lower gestational age (median 25 weeks), birth weight and birth length than pCMV-positive newborns who were not treated (mean of 26 weeks). This could be interpreted as indicating that this subgroup is at a higher risk of a more severe clinical course of pCMV. Short-term outcomes did not differ between pCMV-positive and pCMV-negative newborns in our study, and we have not yet been able to assess long-term outcomes, which could show different results.

It is well established that pCMV is mainly transmitted through breast milk [4,5,6,7,11,36]. In our study, all newborns with pCMV were fed mothers’ milk, either from their mother or donated milk. The milk was either fresh or frozen–thawed and all of the newborns received both types of milk. In our study 22% of the cohort acquired CMV postnatally which may seem unusual. However,, Hu et al. reported in their 2021 meta-analysis of 21 studies including 1920 LBW and preterm infants that the pooled rate of pCMV infection in infants fed untreated and frozen breast milk was 19.3% (95% CI 11.8–29.9%, *p* < 0.01), whereas the pooled rate in infants fed frozen breast milk was 13.5% (95% CI 8.0–22.0%, *p* < 0.01; I^2^ = 46%), which was significantly lower than that observed in infants fed untreated breast milk (overall *p* < 0.01) [4].

Our results were slightly above the pooled rate reported by Hu et al.; however, the reported pCMV infection rate ranged from 11.8% to 29.9%. This variability suggests that differences in the way mother’s milk was handled and administered to preterm infants may have contributed to the observed differences, particularly because pCMV infection was lower in infants fed frozen breast milk. In the same meta-analysis, the pooled CMV infection rate for all infants was 14.4% (95% CI 10.1–20.2%, *p* < 0.01; I^2^ = 40%).

During data collection, we observed that pCMV infection appeared to occur in clusters, regardless of whether the infants were twins. For example, after two infants tested positive for pCMV, several subsequently admitted infants did not acquire the infection, and after some time new positive cases appeared again.

Hypothetically, this pattern could be related to milk handling. If a mother had a limited milk supply, the expressed breast milk may have been fed to the infant immediately, whereas if a mother produced larger volumes, some milk may have been frozen and later thawed, which could have reduced the risk of infection. At present, we do not have another explanation for this observation.

In our study we determined CMV serostatus in the mothers of pCMV+ newborns at the time of diagnosis. We tested blood samples of 10 mothers, and nine mothers of 12 newborns (three twins) were CMV-seropositive. We detected IgM antibodies in combination with high-avidity IgG antibodies in 3/9 mothers, indicating CMV-seroconversion in the mother coinciding with pCMV infection in the newborn. We therefore assume that most of the newborns in the pCMV+ group were infected through breast milk, except for the one newborn of a CMV-seronegative mother, who was likely infected through contact.

Breast milk is a recognized important factor for outcomes of premature newborns [37], so the benefits of feeding premature newborns breast milk outweigh the potential risks of pCMV. How best to feed breast milk to extremely premature newborns is still unknown. Different studies comparing preventive measures for CMV transmission have failed to unequivocally demonstrate the superiority of one measure above the others, but it is clear that feeding extremely premature newborns processed breast milk (pasteurized or frozen–thawed milk) is superior to feeding them unprocessed breast milk [4,38]. Though previous studies have studied CMV viral load kinetics in breast milk [11,39], CMV reactivation and viral kinetics in breast milk of CMV-seropositive mothers remain unpredictable and not well understood. We believe further studies are needed better to characterize CMV reactivation and virolactia in CMV-positive breastfeeding mothers of extremely premature newborns, which could have important clinical consequences.

The most important strengths of our study are its prospective nature and the ability to include the majority (more than 76%) of the extremely premature newborns born in Slovenia between December 2022 and December 2024, namely at the Department of Perinatology, Division of Gynecology, University Medical Centre Ljubljana. Other extremely premature newborns were born at the Department of Perinatology, University Medical Centre Maribor, but were not included in our study. The main limitations are the relatively small number of pCMV-positive newborns, the lack of relevant laboratory parameters at designated timepoints, since we could not systematically order these tests for all of the included newborns, and the inability to follow-up the newborns at the age of 18–24 months to assess the neurodevelopmental impact of pCMV. We also do not know how many clinically well newborns who were not tested for pCMV may nevertheless have had pCMV infection and remained asymptomatic at the median age of 58 days, when we identified the group of newborns with positive pCMV results, or what this might mean for their future development. Addressing this uncertainty warrants further investigation and would provide a more complete understanding of the overall picture of CMV transmission and infection.

The results of our explanatory study show that pCMV is associated with hepatosplenomegaly, thrombocytopenia and leukopenia in extremely premature newborns compared to uninfected controls. It could also be associated with longer length of stay in the NICU, but our study was unable to definitively prove the association. Further studies are therefore needed to determine measures to limit CMV transmission through breast milk in this population.

Further studies with larger cohorts of infants with and without CMV are needed to better discern which pCMV-positive infants require treatment, and to extend follow-up to determine whether pCMV infection is associated with long-term adverse neurodevelopmental outcomes. Nevertheless, pCMV infection in extremely preterm infants remains a challenge for NICU staff, who must determine how best to manage it to optimize and improve outcomes for these vulnerable newborns.

## Figures and Tables

**Table 1:** Newborns’ characteristics at birth—overall and by postnatal CMV status (results of univariate logistic regression).

Characteristic	*n* = 63	pCMV− (*n* = 49)	pCMV+ (*n* = 14)	OR (95% CI)	*p*
Male; *n* (%)	35 (56)	25 (51)	10 (71)	2.4 (0.66; 8.7)	0.183
Median (IQR) gestational age [weeks]	25 (24–27)	26 (24–27)	25 (24–26)	0.75 (0.5; 1.12)	0.155
Median (IQR) birth weight [g]	750 (650–950)	780 (660–950)	700 (590–910)	1 (1; 1)	0.268
Median (IQR) length at birth [cm]	33 (32–35)	33 (32–36)	32 (30–33)	0.8 (0.65; 0.99)	0.042
Median (IQR) head circumference [cm]	23.5 (22–24.5)	23.5 (22.5–24.5)	22.8 (21.5–23.5)	0.74 (0.53; 1.03)	0.077
SGA; *n* (%)	12 (19)	8 (17)	4 (29)	2 (0.5; 8)	0.327
Median (IQR) Apgar 1 min	6 (4–7)	6 (4–8)	6 (4–7)	1 (0.76; 1.3)	0.976
Median (IQR) Apgar 5 min	7 (6–8)	7 (6–8)	7 (6–8)	0.89 (0.65; 1.22)	0.474
Breastfed; *n* (%)	59 (94)	45 (92)	14 (100)		0.149 *

* LR test.

**Table 2:** Clinical characteristics and treatment outcomes—overall and by postnatal CMV status (results of univariate logistic regression).

Characteristic	*n* = 63	pCMV− (*n* = 49)	pCMV+ (*n* = 14)	OR (95% CI)	*p*
Treatment with dexamethasone; *n* (%)	32 (52)	22 (46)	10 (71)	2.95 (0.81; 10.74)	0.1
Treatment with diuretics; *n* (%)	26 (41)	17 (35)	9 (64)	3.39 (0.98; 11.73)	0.054
Use of vasopressors; *n* (%)	4 (6)	4 (8)	0 (0)		0.149 *
Respiratory support; *n* (%)	47 (75)	35 (71)	12 (86)	2.4 (0.47; 12.13)	0.29
NIV	25 (53)				
MV	22 (47)				
Median (IQR) FiO_2_	0.3 (0.2–0.4)	0.3 (0.2–0.4)	0.3 (0.2–0.5)	8.26 (0.32; 210.03)	0.201
Splenomegaly; *n* (%)	6 (10)	1 (2)	5 (36)		<0.001 *
Hepatomegaly; *n* (%)	4 (6)	0 (0)	4 (29)		<0.001 *
Hepatosplenomegaly; *n* (%)	4 (6)	0 (0)	4 (29)		<0.001 *
Jaundice; *n* (%)	2 (1.6)	0 (0)	1 (7.1)		0.08 *
SLS; *n* (%)	2 (3)	1 (2)	1 (7)	3.62 (0.21; 61.82)	0.375
PDA; *n* (%)	24 (38)	16 (33)	8 (57)	2.75 (0.82; 9.27)	0.103
Bacterial sepsis; *n* (%)	6 (10)	6 (12)	0 (0)		0.074
PVL; *n* (%)	4 (6)	3 (6)	1 (7)	1.18 (0.11; 12.31)	0.89
BPD; *n* (%)	48 (76)	35 (71)	13 (93)	5.2 (0.62; 43.6)	0.129
					
BPD stage; *n* (%)					
Mild	17 (37)	11 (33)	6 (46)		
Moderate	22 (48)	18 (55)	4 (31)	0.41 (0.09; 1.77)	0.231
Severe	7 (15)	4 (12)	3 (23)	1.38 (0.23; 8.30)	0.728
ROP; *n* (%)	35 (56)	25 (51)	10 (71)	2.4 (0.66; 8.7)	0.183
					
Severe ROP stage; *n* (%)	11 (31.4)	9 (36)	2 (20)	0.44 (0.08; 2.56)	0.364
NEC; *n* (%)	5 (8)	4 (8)	1 (7)	0.87 (0.09; 8.43)	0.901
Severe NEC; *n* (%)	3 (60)	3 (75)	0 (0)		0.135 *
					
IVH 3/4; *n* (%)	4 (6)	4 (8)	0 (0)		0.149 *
					
					
					
Median (IQR) LOS	73 (59–107)	71 (58–94)	111 (66–142)	1.02 (1; 1.04)	0.014
TEOAE; *n* (%)					
Responsive bilaterally	42 (72)	34 (76)	8 (62)	1	
Nonresponsive unilaterally	7 (12)	4 (9)	3 (23)	3.19 (0.59; 17.16)	0.177
Nonresponsive bilaterally	9 (16)	7 (16)	2 (15)	1.21 (0.21; 6.99)	0.828
Death; *n* (%)	2 (3)	2 (4)	0 (0)		0.312 *

* LR test.

**Table 3:** Laboratory results of newborns—overall and by postnatal CMV status (results of univariate logistic regression).

Characteristic	All	pCMV−	pCMV+	OR (95% CI)	*p*
Mean ± SD platelet count [×109/L]	317.5 (129–427)	342 (208–455)	116 (104–218)	0.99 (0.99; 1)	0.006
Mean ± SD leukocyte count [×109/L]	16.2 (10.3–20)	17.7 (11.9–24.4)	9.3 (7.9–12)	0.78 (0.65; 0.93)	0.006
	*n* = 50	*n* = 37	*n* = 13		
Mean ± SD neutrophil count [×109/L]	8.1 (5.2–11.3)	8.5 (6.4–13)	3.2 (1.3–10.7)	0.88 (0.75; 1.03)	0.104
	*n* = 45	*n* = 34	*n* = 11		
Median (IQR) CRP [μmol/L]	0 (0–12)	0 (0–1)	11 (0–36)	1.01 (0.99; 1.03)	0.576
	*n* = 46	*n* = 36	*n* = 10		

## Data Availability

The original contributions presented in this study are included in the article. Further inquiries can be directed to the corresponding author.

## Abbreviations

The following abbreviations are used in this manuscript:

CMV	Cytomegalovirus
cCMV	Congenital cytomegalovirus infection
pCMV	Postnatal cytomegalovirus infection
NEC	Necrotizing enterocolitis
BPD	Bronchopulmonary dysplasia
ROP	Retinopathy of prematurity
SNHL	Sensory neural hearing loss
IgM	Immunoglobulin M
IgG	Immunoglobulin G
NICU	Neonatal intensive care unit
UMC	University Medical Center
AST	Aspartate aminotransferase
ALT	Alanine aminotransferase
γ-GT	Gamma-glutamyl transferase
CRP	C-reactive protein
SGA	Small for gestational age
PVL	Periventricular leukomalacia
IVH	Intraventricular hemorrhage
TEOAE	Transitory evoked otoacoustic emissions
LOS	Length of stay
SD	Standard deviation
IQR	Interquartile range
NIV	Non-invasive ventilation
MV	Mechanical ventilation
FiO2	Fraction of inspired oxygen
SLS	Sepsis-like syndrome
PDA	Patent ductus arteriosus
